# Enhancement of Growth, Antioxidant Activity, and Immunity in Nile Tilapia (*Oreochromis niloticus*) Through Recombinant *Bacillus subtilis* Expressing L-Gulonolactone Oxidase

**DOI:** 10.3390/antiox14010050

**Published:** 2025-01-04

**Authors:** Jirawadee Kaewda, Surintorn Boonanuntanasarn, Papungkorn Sangsawad, Pimpisut Manassila, Chatsirin Nakharuthai

**Affiliations:** School of Animal Technology and Innovation, Institute of Agricultural Technology, Suranaree University of Technology, 111 University Avenue, Muang, Nakhon Ratchasima 30000, Thailand; m6500115@g.sut.ac.th (J.K.); surinton@sut.ac.th (S.B.); papungkorn@sut.ac.th (P.S.); m6500122@g.sut.ac.th (P.M.)

**Keywords:** L-gulonolactone oxidase, antioxidant, *Bacillus subtilis*, antagonistic activity, Nile tilapia, *Streptococcus agalactiae*

## Abstract

Due to its lack of the L-gulonolactone oxidase (*GULO*) enzyme, Nile tilapia is unable to synthesize vitamin C; thus, it requires an adequate level of exogenous vitamin C in its diet. To enhance antioxidant properties and vitamin C-related effects, we employed recombinant technology to integrate the *GULO*-encoding gene into the *Bacillus subtilis* chromosome. In this study, fish were divided into four groups: those fed with a basal diet (CON), a basal diet + vitamin C (VC), a basal diet + wild-type *B. subtilis* (BS), and a basal diet + recombinant *B. subtilis* (BS+GULO). After 90 days of the feeding trial, the BS+GULO groups showed the highest improvements in final weight, weight gain, specific growth rate, average daily gain, and relative growth rate. The VC, BS, and BS+GULO groups exhibited increased total immunoglobulin and lysozyme activity; however, only the VC and BS+GULO groups showed elevated alternative complement 50 levels, phagocytic activity and improved antioxidant parameters compared to the control. HPLC and qRT-PCR analyses revealed elevated serum vitamin C and intestinal *GULO* mRNA levels in the BS+GULO group. A challenge test showed increased pro-inflammatory gene expression and immune response against *S. agalactiae* in the BS+GULO group, indicating improved antagonistic activity over wild-type *B. subtilis*.

## 1. Introduction

In recent decades, the production of Nile tilapia has steadily shifted towards intensive culture systems [1]. However, the advancement of intensive culture has led to the deterioration of water quality, facilitating the proliferation of pathogens in aquatic environments [2]. In such situations, coupled with the impact of climate change, fish are more susceptible to stress, leading to impaired growth performance and a weakened immune system. To address this issue, fish farmers have prioritized fish health maintenance by implementing effective management practices, supplying high-quality nutritional feed, and administering immunostimulants [3,4]. Among the immunostimulant agents, probiotics *Bacillus subtilis* and vitamin C have attracted research interest for application in intensive culture systems [5,6,7,8,9,10,11] The probiotic *B. subtilis* is one of the most commonly used dietary supplements in various fish species owing to its numerous positive effects on the gut microbiota, growth performance, disease resistance, health status of aquatic animals, and water quality [5,6,7,12]. Moreover, it is generally recognized as safe for humans, animals, and the environment under specified conditions of use. Additionally, it is widely considered an ideal bacterial factory for producing heterologous proteins [8,9].

In modern fish farming, vitamin C is a crucial exogenous micronutrient and immunostimulant in aquafeed, as natural levels are often insufficient to support normal body functions in fish, particularly under intensive aquaculture conditions. Due to vitamin C’s pivotal function as an enzyme cofactor, it plays a crucial role in facilitating many physiological processes that involve biosynthesis, protein metabolism [10], iron metabolism [13], lipid metabolism [14], immune response [15], stress [16], and physiological antioxidant activity [17,18]. In fish, vitamin C deficiency has various adverse consequences, including impaired growth and survival rate, increased susceptibility to stress, depressed immune status, reduced reproductive performance, skeletal alterations, impaired collagen formation, slow wound healing, and anemia [19,20,21,22].

On the other hand, adequate vitamin C intake has been widely shown to have beneficial effects on the growth and health of fish. For example, dietary supplementation with the optimal level of vitamin C markedly improved growth performance [22,23,24] and serum antioxidant activities [22]. It also enhanced several immune responses, including phagocytic activity, phagocytic index, alternative complement activity (ACH_50_), and lysozyme activity (LZM) [23,25,26]. In addition, dietary vitamin C increment has also been proven to enhance the proliferation of spermatogonia and hematocrit value in Japanese eel broodstock (*Anguilla japonica*) [27].

In aquaculture conditions, vitamin C is naturally derived from plants found in aquatic environments. However, in intensive commercial operations, natural plant-based food sources are usually inadequate to meet the required amounts for fish. Moreover, more advanced teleosts, including Nile tilapia, are incapable of synthesizing vitamin C de novo due to a lack/mutation of the L-gulonolactone oxidase (*GULO*), an enzyme necessary for the last step of ascorbic acid biosynthesis [28]. In contrast, amphibians, reptiles, mammals (such as mice, sheep, and dogs), birds, chickens, primitive lobe-finned fish, cartilaginous fish species (such as shark species and white sturgeon), and almost all plants possess the ability to synthesize vitamin C due to the presence of the functional *GULO* gene [29,30,31].

As a result, more advanced teleost species must obtain vitamin C through dietary supplementation to ensure their optimum growth and health, especially in intensive culture conditions where limited natural foods are available [32]. Unfortunately, the stability of vitamin C as a dietary component often makes it inadequate at proper levels for aquatic animals due to its rapid oxidation. The loss of vitamin C is accelerated in inappropriate environmental conditions during the commercial manufacturing process of aquafeed, storage, handling, and feeding. The loss rate depends on various factors, including temperature, oxygen, UV irradiation, light, pH levels, and transition metal ions [33,34].

Various approaches have emerged to ensure that animals receive a sufficient amount of vitamin C and to enhance its stability and bioavailability. They include the shielding of vitamin C through encapsulation [35], the development of chemical vitamin C derivatives [36], genomic integration of L-gulonolactone oxidase [30,37], utilization of exogenous 2-keto-L-gulonic acid supplementation [38], and so on. In this study, we aimed to construct a recombinant probiotic *B. subtilis* expressing *GULO* from the red junglefowl (*G. gallus*) to re-establish the ascorbic acid pathway in Nile tilapia and evaluate its potential as a dietary supplement for Nile tilapia. Since the ascorbate biosynthesis pathway, starting with D-glucose-1-phosphate as the initial precursor and progressing until L-gulonate, is conserved in all animal species [39], it may be possible to re-establish this pathway by integrating the *GULO* gene into probiotic *B. subtilis* using recombinant probiotic technology.

The advancement of recombinant technology has facilitated the production of heterologous proteins using potential probiotics as expression systems. Recombinant probiotics offer a promising approach to delivering the specific traits and functionalities of heterologous proteins. Among these, the genus *Bacillus* has gained recognition as a reliable biofactory for producing heterologous proteins, serving both basic research and industrial applications [40,41,42]. Employing *Bacillus* spp. presents numerous advantages, including their capacity for rapid and high-yield product synthesis, ease of genetic modification, and suitability for the expression and delivery of target genes.

In our previous study, we isolated and characterized the potential probiotic *B. subtilis* B29 from the intestinal microbiota of Nile tilapia based on its biological functions [42]. Our investigation elucidated its advantageous properties, emphasizing antagonistic activity as the main criterion for selection, along with bile salts and pH tolerance, protease-producing capacity, antibiotic susceptibility, and results from pathogenicity tests. It is noteworthy that the probiotic *B. subtilis* B29 exhibited antagonistic activity against the three primary pathogenic bacteria in Nile tilapia, namely *Aeromonas hydrophila*, *Streptococcus iniae*, and *Streptococcus agalactiae*, with higher efficacy against *S. agalactiae*.

In Thailand, *S. agalactiae*, a Gram-positive pathogenic bacterium, is frequently encountered in Nile tilapia. This bacterium causes the disease known as ‘streptococcosis’, which is characterized by several clinical symptoms in Nile tilapia, including unilateral or bilateral exophthalmia, erratic swimming, hemorrhaging in both external and internal organs, and septicemia [43,44]. Currently, it is recognized for causing significant mortality, typically occurring over a brief timeframe, particularly in intensive Nile tilapia farms [45]. Therefore, the application of recombinant probiotic *B. subtilis* expressing *GULO* may provide a possible alternative option to achieve the combined effect of probiotic *B. subtilis* and vitamin C supplementation.

## 2. Materials and Methods

### 2.1. Construction of Recombinant Probiotic B. subtilis Expressing GULO

#### 2.1.1. Primer Design, RNA Isolation, and cDNA Synthesis

Specific primers were deliberately designed to amplify the full-length *GULO* cDNA of *G. gallus* (accession no. XM_015285218), published in the GenBank database (http://www.ncbi.nlm.nih.gov (accessed on 1 March 2022). The forward primer (H-B-*GULO*F) containing *Hind*III and *Bam*HI restriction sites was designed from the start codon (ATG), while the reverse primer (H-X-*GULO*R) was designed prior to the stop codon followed by *Xho*I and *Hind*III, respectively. Total RNA was extracted from the kidney of *G. gallus* using the TRlzol reagent (Gibco BRL, Gaithersburg, MD, USA) in accordance with the manufacturer’s instructions in order to amplify full-length *GULO* cDNA. Briefly, 100 mg of the kidney was homogenized using mini-beadbeater-16 (Thermo Fisher Scientific, Waltham, MA, USA) and subsequently extracted using the conventional phenol–chloroform method with some modifications. To eliminate genomic DNA, the dissolved total RNA was treated with RQ1 RNase-Free DNase (Promega Corporation, Madison, WI, USA). The three intact bands of RNA were visualized on agarose gel electrophoresis stained with SafeRed nucleic acid staining solution (Vivantis Technologies Sdn Bhd., Selangor, Malaysia). The Nanodrop 2000™ spectrophotometer (Thermo Fisher Scientific) was used to measure the quantity and quality of RNA. After that, first-strand cDNA was synthesized using the ImProm-II™ Reverse Transcription System kit (Promega) and kept at −20 °C in a freezer until use.

#### 2.1.2. Cloning of the Full-Length GULO cDNA of *G. gallus* into pGEM^®^T-Easy

To construct recombinant *B. subtilis* expressing *GULO*, the full-length *GULO* cDNA was amplified using gene-specific primers (Table 1). PCR was performed under the following conditions: 95 °C for 3 min, then 40 cycles at 95 °C for 30 s, 59.9 °C for 30 s, 72 °C for 3 min, followed by 72 °C for 5 min. The PCR product was purified using FavorPrep™ GEL/PCR Purification Kit (Farvogen^®^ Biotech Corp, Ping Tung, Taiwan) and subsequently sequenced using Macrogen sequencing service (Macrogen Inc., Seoul, Republic of Korea) with forward (H-B-*GULO*F) and reverse (H-X-*GULO*R) primers to confirm the nucleotide and amino acid sequence accuracy of the PCR product. After that, the purified PCR was ligated into a pGEM^®^T-Easy plasmid (Promega) under the conditions described in the manufacturer’s protocol. Finally, the ligation product was transformed into 100 µL of *E. coli* DH5α competent cells using the heat shock method. Transformed bacteria containing plasmid DNA exhibiting white-colored colonies were selected for further analyses, which included colony PCR screening, digestion with restriction enzymes (*Hi*ndIII and *Bam*HI), and confirmation of the insert DNA via sequencing (Macrogen, Republic of Korea).

#### 2.1.3. Transformation of GULO Plasmid into Probiotic *B. subtilis* via Electroporation

The positive *GULO* plasmids from 2.1.2. and the pBES expression vector (Takara Bio USA, Inc., San Jose, CA, USA) were double-digested with *Hind*III and *Bam*HI and purified before ligation. The ligation reaction was then transformed into *E. coli* DH5α competent cells, and the accuracy of the nucleotide and amino acid sequence was confirmed by sequencing. The electroporation method was used to transform the pBES*GULO* plasmid into probiotic *B. subtilis* competent cells, which were prepared following the method of Xue et al. [46] with minor modifications. To identify the positive clones, the transformants were spread onto a Luria–Bertani (LB) plate with 100 μg mL^−1^ of kanamycin and incubated for 16–18 h at 37 °C, and the apparent clones were proved by colony PCR, double restriction enzyme digestion, and sequencing to confirm nucleotide and amino acid sequence accuracy.

#### 2.1.4. Western Blotting Analysis

Prior to commencing the feeding experiment, the presence of secreted *GULO* produced from probiotic *B. subtilis* was confirmed through the Western blot method, as in our previous study [42].

### 2.2. Ethics Statement

All animal experiments were conducted in compliance with the regulations and approved by the Ethics Committee of Suranaree University of Technology (SUT), Animal Care and Use Committee (approval no. SUT-IACUC-0012/2023).

### 2.3. The Effect of Dietary Recombinant Probiotic B. subtilis Expressing GULO Supplementation in Normal Fish

#### 2.3.1. Experimental Design

This experiment was conducted on four groups, each with three replication tanks and ten individual fish, using a completely randomized design. A total of 120 healthy Nile tilapia were divided across twelve 700 L fiber tanks containing clean fresh water with an aeration system. After a 2-week acclimatization period, the fish were fed ad libitum twice daily with the following four experimental diets: a commercial diet + 0.85% NaCl (CON) (*n* = 30), a commercial diet supplemented with vitamin C (VC) (*n* = 30), a commercial diet supplemented with wild-type *B. subtilis* (BS) (*n* = 30), and a commercial diet supplemented with recombinant *B. subtilis* expressing *GULO* (BS+GULO) (*n* = 30). A concentration of 0.35 mL L^−1^ of 2-phenoxyethanol was administered as an anesthetic during all sampling processes.

#### 2.3.2. Diet Preparation

Before commencing this experiment, both wild-type isolated *B. subtilis* and the recombinant probiotic *B. subtilis* expressing *GULO* were proliferated, aliquoted, and stored in glycerol stocks at −80 °C until use. For the preparation of each experimental diet, aliquots of wild-type isolated *B. subtilis* and probiotic *B. subtilis* expressing *GULO* were separately inoculated into LB broth and LB broth containing kanamycin, respectively. The inoculated cultures were then incubated in an incubator shaker at 37 °C for 18–24 h. After harvesting each bacterial suspension by centrifugation at 5000× *g* for 5 min, the bacterial pellets were washed twice with sterile 0.85% NaCl and then resuspended in the same solution to adjust the concentration to 1 × 10^8^ colony-forming units (CFU mL^−1^). Briefly, 1 × 10^8^ CFU mL^−1^ of wild-type *B. subtilis* and recombinant *B. subtilis* expressing *GULO* in a total volume of 100 mL were separately mixed with 500 g of commercial floating pellet diet and slowly stirred. The control diet was prepared using 100 mL of 0.85% sterile NaCl per 500 g of the same diet. The mixed diets were coated with 2.5% (*v*/*w*) sterile squid oil and air-dried at room temperature for 3 h. After that, the experimental diets were aliquoted for daily use before being stored at 4 °C until feeding the fish. In the vitamin C supplementation group, 500 mg kg^−1^ of vitamin C (Stay C-35™, F. Hoffmann-La Roche, Basel, Switzerland) was prepared by mixing in the fish diet using the same method described above for the probiotic supplementation. The major chemical compositions of a commercial diet were analyzed according to the standard method of the Association of Official Analytical Chemists (AOAC) (1990) [47], which includes 30% crude protein, 12% moisture, 8% fiber, and 3% fat. No significant difference was observed among the experimental diets (*p* ≥ 0.05).

#### 2.3.3. Growth Performance

The individual body weight and length of fish from each tank were measured at 0, 30, and 90 days to assess the growth performance indices, namely the weight gain (WG), specific growth rate (SGR), average daily gain (ADG), feed conversion ratio (FCR), protein efficiency ratio (PER), and relative growth rate (RGR) (*n* = 10 fish per group). These indices were calculated as follows:

$$\begin{array}{l} \mathrm{WG}=\mathrm{FW}-\mathrm{IW}\\ \mathrm{ADG}\;(g/\mathrm{day})=(\mathrm{FW}-\mathrm{IW})/\mathrm{experimental}\;\mathrm{days}\\ \mathrm{SGR}\;(\%/\mathrm{day})=100\times [(\ln\mathrm{FW}-\ln\mathrm{IW})/\mathrm{experimental}\;\mathrm{days}]\\ \mathrm{FCR}=\mathrm{dry}\;\mathrm{feed}\;\mathrm{fed}/\mathrm{wet}\;\mathrm{weight}\;\mathrm{gain}\\ \mathrm{PER}=\mathrm{Weight}\;\mathrm{gain}/\mathrm{protein}\;\mathrm{intake}\\ \mathrm{RGR}\;(\%)=(\mathrm{Final}\;\mathrm{weight}-\mathrm{Initial}\;\mathrm{weight})/\mathrm{Initial}\;\mathrm{weight}\times 100 \end{array}$$

where FW is final weight, and IW is initial weight.

#### 2.3.4. Determination of Vitamin C in Nile Tilapia Serum Using HPLC Analysis

To analyze the concentration of vitamin C in experimental fish, the serum was collected from the experimental fish after 90 days of the feeding trial (*n* = 3). The HP 1100 series reversed-phase high-performance liquid chromatography (HPLC) system (Agilent Technologies, Waldbronn, Germany) with a C18 HPLC column, 5 µm, 250 × 4.0 mm was used in this experiment according to the method described by Pitaksong et al. [48]. The mobile phase was used with a flow rate of 0.8 mL min^−1^. The serum was centrifuged at 10,000 rpm for 10 min to remove debris. The clear supernatant was filtered through a 0.45 μm syringe filter. Twenty microliters of clear filtrate were injected into the HPLC system. High-purity vitamin C (Sigma, St. Louis, MO, USA) was used as the reference standard for quantifying vitamin C in fish serum. Each experiment was conducted in triplicate.

#### 2.3.5. Immune Parameters

At 30 and 90 days of the trial, serum LZM activity [49], serum total immunoglobulin (total Ig) concentration [42], and serum ACH_50_ activity [50] were assessed (*n* = 3) in a slightly modified manner. The optical density of each sample was measured using an absorbance microplate reader Epoch BioTek instruments (Agilent Technologies). In addition, at 90 days of the trial, the phagocytic activity of peripheral blood leukocytes (PBLs) was determined (*n* = 3) by appropriately modifying the methods described by Puangkaew et al. [51].

#### 2.3.6. Serum Antioxidant Enzyme Activities

The activity of catalase (CAT), total antioxidant capacity (TAC), superoxide dismutase (SOD), Glutathione Peroxidase (GSH-Px), and malondialdehyde (MDA) in the serum of the experimental fish (*n* = 3) at 90 days of the trial were measured using a commercial kit (Abbkine Corporation, Atlanta, GA, USA) according to the manufacturer’s recommended protocol.

#### 2.3.7. Expression of GULO mRNA in Normal Fish via qRT-PCR

To construct cDNA plasmid standards for qRT-PCR, cloning of each target gene of interest was performed to evaluate the mRNA expression level of Nile tilapia (*n* = 3) at the end of the feeding trial. The primer sets for qRT-PCR analysis used in this study are shown in Table 1. PCR products of the expected size were purified using a FavorPrep GEL/PCR Purification Kit according to the manufacturer’s instructions. Purified DNA was cloned into pGEM^®^ T-Easy plasmids, and the positive clones were screened as described in 2.1.2. Finally, the selected plasmids were sequenced by Macrogen, Inc. (Seoul, Republic of Korea) and stored at −20 °C for use as a standard for qRT-PCR. One microliter of first-strand cDNAs was subjected to qPCR analysis (in triplicate) using a CFX Opus Real-Time PCR System machine (Bio-Rad, Hercules, CA, USA). Each reaction was performed in a final volume of 10 µL containing 1 µL cDNA, 5 µL thunderbird SYBR^®^ qPCR master mix (TOYOBO, Osaka, Japan), 2 µL dH_2_O, and 1 µL each of specific primer, as shown in Table 1. The PCR conditions were as follows: 95 °C for 3 min, followed by 40 cycles of 95 °C for 30 s and 55–59 °C for 30 s. DNA melting curve analysis was used to verify the specificity of the primers. The internal reference for data normalization was the β-actin mRNA. The mRNA level was quantitatively analyzed, as described in Boonanuntanasarn et al. [52].

### 2.4. The Effect of Dietary Supplementation with Probiotic B. subtilis Expressing GULO After a Challenge with S. agalactiae in Nile Tilapia

#### 2.4.1. Experimental Design

To determine the immune response of Nile tilapia following injection with *S. agalactiae*, a total of 60 fish (*n* = 15) were used in this experiment after one month of the feeding trial. The fish were distributed into twelve 500 L fiber tanks, with three replication tanks per diet group, each containing five individual fish.

#### 2.4.2. Preparation of *S. agalactiae* and Challenge Test

The virulent strain of *S. agalactiae*, which was previously isolated by our research group [53], was used in the challenge experiment after the feeding trial for 30 days. The single colony of *S. agalactiae* was inoculated in tryptic soy broth (Merck KGaA, Darmstadt, Germany) at 37 °C for 16–18 h with shaking. The concentration of *S. agalactiae* was adjusted to 1 × 10^8^ CFU mL^−1^ with an optical density at 600 nm of 1.0. The experimental fish were intraperitoneally injected (i.p.) with a 1 × 10^8^ CFU mL^−1^ suspension of live *S. agalactiae* in a volume of 0.1 mL per 100 g of fish body weight. This concentration represents 10% of the LD50 (1 × 10^9^ CFU/mL), providing a sub-lethal dose that avoided acute or immediate mortality.

#### 2.4.3. Immune Parameters and Expression of Pro-Inflammation Genes in Challenged Fish

After the challenge, the liver, spleen, and serum of injected fish were collected at 0 h, 6 h, 12 h, 24 h, and 48 h (*n* = 3 fish per group, per time point; total *n* = 60 fish). The serum samples were then analyzed for immune parameters (LZM, total Ig, and ACH_50_), as outlined in Section 2.3.5. Pro-inflammatory gene expressions (CC chemokine and tumor necrosis factor alpha (TNFα)) in the liver and spleen of challenged fish were assessed using qRT-PCR, as previously described.

### 2.5. Statistical Analysis

The statistical analyses using the SPSS software ver.25 (SPSS Inc., Chicago, IL, USA). The data were analyzed using a one-way analysis of variance followed by the post hoc Tukey’s test to assess the significance of differences between the groups. A paired-sample *t*-test was conducted to evaluate the difference between 30 and 90 days after the feeding trial within immune parameters and the expression of *GULO* mRNA. The difference between groups in comparative experiments was determined by statistical significance at *p* < 0.05.

## 3. Results

### 3.1. Construction of Recombinant B. subtilis Expressing GULO and Western Blot Analysis

To determine the accuracy of the nucleotide sequence and the correct in-frame insertion, the sequence of the *GULO* gene was confirmed via sequencing. The result demonstrated that the full-length cDNA encoding the *GULO* gene of *G. gallus* comprised an 1323 bp open reading frame (ORF) and the predicted amino acid sequence of *GULO* contained 440 amino acid residues (Figure S1). In addition, the double restriction enzyme (*Bam*HI and *Hind*III) could identify the insertion of the *GULO* gene into cloning and expression vectors (Figure S2). Before initiating the feeding trial, the presence of the *GULO* produced by probiotic *B. subtilis* was confirmed through Western blot analysis, revealing its molecular weight (Mw) to be approximately 50 kDa (Figure S3).

### 3.2. Growth Performance

The results of growth performance in Nile tilapia fed with experimental diets are presented in Table 2. On day 30 of the feeding trial, there were no significant differences (*p* ≥ 0.05) in growth performance parameters among the experimental diets, except for the PER in wild-type *B. subtilis* and recombinant *B. subtilis* expressing *GULO* groups. The results showed that the PER of these groups was significantly increased (*p* < 0.05) compared with the vitamin C and control groups. Interestingly, at day 90 of the feeding trial, the recombinant *B. subtilis* expressing *GULO* group exhibited the highest positive effect on FW, WG, FCR, ADG, SGR, and RGR in comparison to the other groups, whereas supplementation with vitamin C and wild-type *B. subtilis* improved only FW, WG, and ADG (*p* < 0.05).

### 3.3. Determination of Vitamin C in Nile Tilapia Serum Using HPLC Analysis

Following a 90-day feeding trial, the assessment of vitamin C levels in the serum of Nile tilapia was performed using HPLC analysis. The findings indicated a significant elevation in the serum vitamin C levels in the recombinant *B. subtilis* expressing *GULO* group compared to the control and wild-type groups. However, these levels were also lower than those observed in the vitamin C group (Table 3).

### 3.4. Expression of GULO mRNA in Normal Fish by qRT-PCR

**Figure 1:**
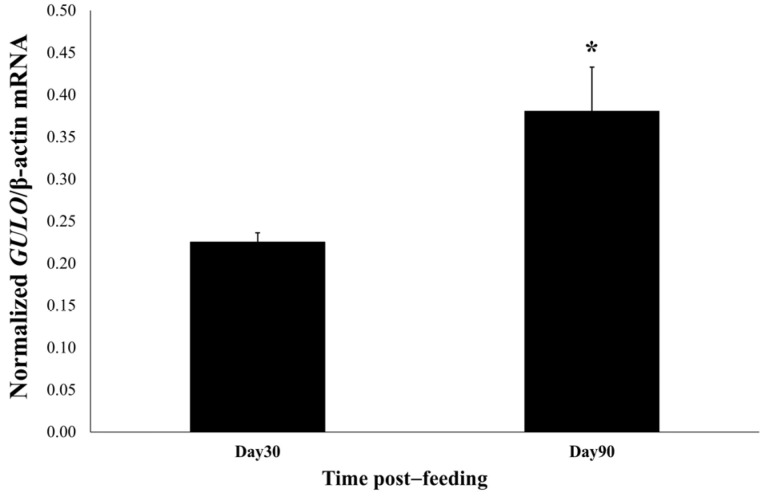
The expression levels of *GULO* mRNA in the intestine of Nile tilapia were compared between those fed experimental diets at 30 days and those at 90 days of the feeding trial (*n* = 3). The asterisk indicates significant statistical differences (*p* < 0.05).

To provide supporting evidence regarding the existence of *GULO* in Nile tilapia, qRT-PCR was carried out to quantify *GULO* mRNA expression in the intestines of the experimental fish at days 30 and 90 of the feeding trial. The *GULO* mRNA expression level was detected only in the recombinant *B. subtilis* expressing *GULO* group at both time points. Moreover, on day 90, the expression level of *GULO* mRNA exhibited a significant increase compared to day 30, as shown in Figure 1.

### 3.5. Immune Responses

**Figure 2:**
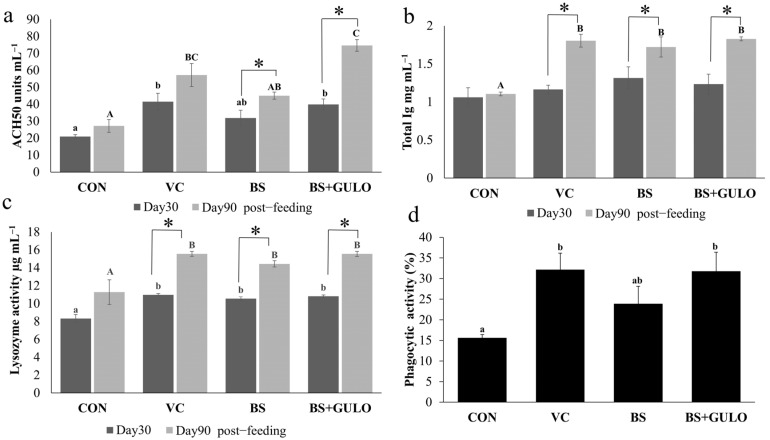
Immune parameters of Nile tilapia fed experimental diets for 30 days and 90 days of a feeding trial (*n* = 3). ACH50 (**a**); total Ig (**b**); LZM (**c**). Bars with asterisks indicate significant differences between day 30 and day 90 of the feeding trial, whereas bars labeled with different lowercase letters denote significant differences for day 30 of the feeding trial, and bars labeled with uppercase letters indicate significant differences for day 90 of the feeding trial, respectively (*p* < 0.05). Phagocytic activity (%) of phagocytic cells in PBLs of Nile tilapia fed experimental diets for 90 days of the feeding trial (**d**). Bars with different letters indicate significant differences (*p* < 0.05). Abbreviations: a basal diet (CON); a basal diet + vitamin C (VC); a basal diet + wild-type *B. subtilis* (BS); and a basal diet + recombinant *B. subtilis* (BS+GULO).

On day 30 and day 90 of the feeding trial, the vitamin C- and recombinant *B. subtilis* expressing *GULO* groups showed significant increases in ACH_50_ levels compared to the control groups. Interestingly, only the wild-type *B. subtilis*- and recombinant *B. subtilis* expressing *GULO* groups demonstrated significant increases in ACH_50_ levels between days 30 and 90 (Figure 2a). Regarding total Ig levels, no significant difference was observed among the experimental groups on day 30 of the feeding trial. However, by day 90, the vitamin C, wild-type *B. subtilis*, and recombinant *B. subtilis* expressing GULO groups showed significant increases in total Ig levels compared to the control group, with levels rising significantly between days 30 and 90 (Figure 2b). In terms of LZM, the vitamin C, wild-type *B. subtilis*, and recombinant *B. subtilis* expressing GULO groups showed significant increases on days 30 and 90 compared to the control group, with levels rising significantly between days 30 and 90 (Figure 2c). Finally, only the vitamin C and recombinant *B. subtilis* expressing *GULO* groups exhibited significant increases in phagocytic activity compared to the control groups (Figure 2d).

### 3.6. Antioxidant Enzyme Parameters in Nile Tilapia Serum

At the end of the 90-day feeding trial, a significant increase in serum levels of SOD, CAT, TAC, and GSH-Px, along with a decrease in MDA levels, was observed in the vitamin C and recombinant *B. subtilis* expressing *GULO* groups compared to the control groups, respectively (Table 4).

### 3.7. Immune Parameter After S. agalactiae Injection

**Figure 3:**
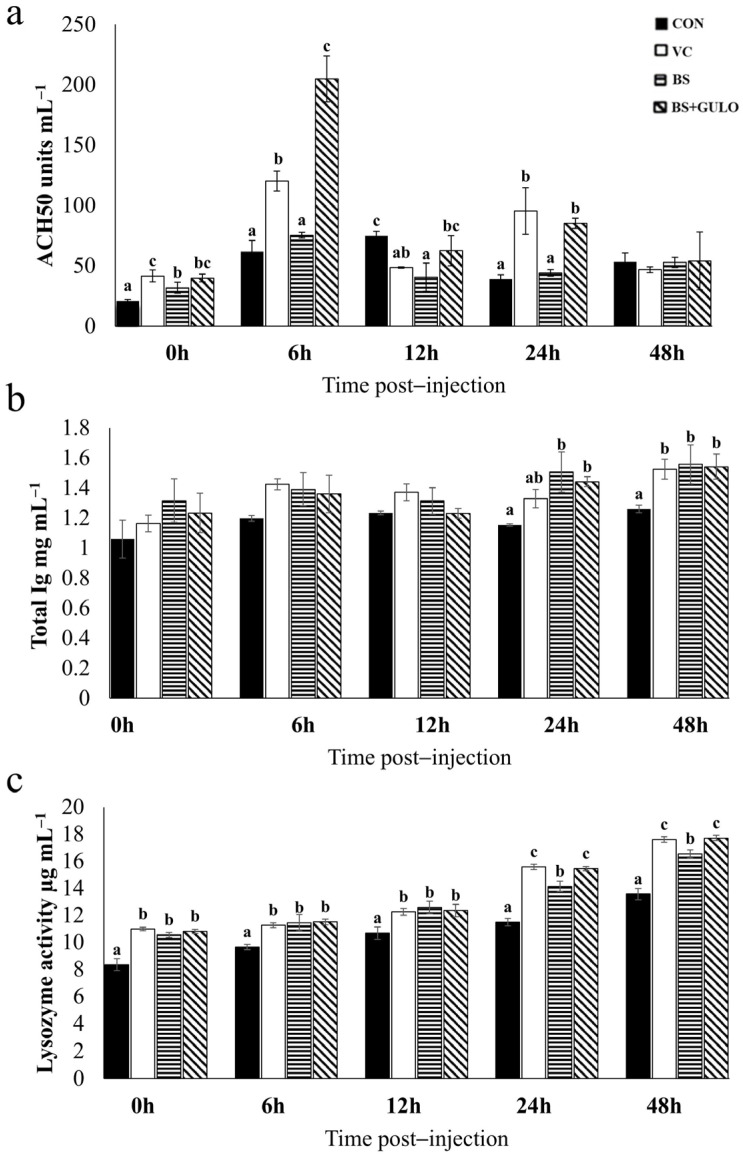
Immune parameters of Nile tilapia in response to *S. agalactiae* at different time points following the 30-day feeding trial (*n* = 3). ACH_50_ (**a**); total Ig (**b**); LZM (**c**). Bars with different letters indicate significant differences (*p* < 0.05). Abbreviations: a basal diet (CON); a basal diet + vitamin C (VC); a basal diet + wild-type *B. subtilis* (BS); and a basal diet + recombinant *B. subtilis* (BS+GULO).

After the challenge test, a rapid upregulation of ACH_50_ was observed at 6 h post-injection in the vitamin C and recombinant *B. subtilis* expressing *GULO* groups (Figure 3a). In the case of total Ig, significant increases were observed at 24 and 48 h post-injection, but only in the wild-type *B. subtilis* and recombinant *B. subtilis* expressing *GULO* groups compared to the control group (Figure 3b). Regarding LZM, higher upregulation was observed in all experimental groups compared to the control group at all time points (Figure 3c).

### 3.8. Pro-Inflammatory Gene Expression After S. agalactiae Injection

**Figure 4:**
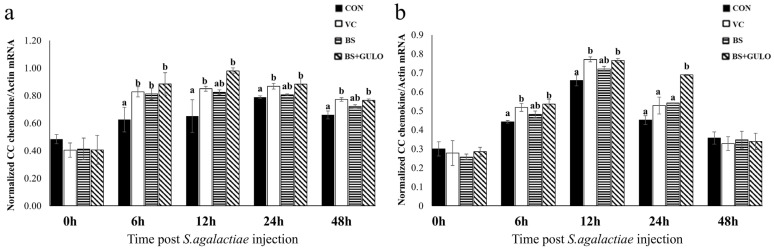
Quantitative real-time PCR analysis of CC chemokine expression in the spleen (**a**) and liver (**b**) of Nile tilapia in response to *S. agalactiae* at different time points following the 30-day feeding trial (*n* = 3). The different letters on each bar indicate significant differences at *p* < 0.05. Abbreviations: a basal diet (CON); a basal diet + vitamin C (VC); a basal diet + wild-type *B. subtilis* (BS); and a basal diet + recombinant *B. subtilis* (BS+GULO).

After the challenge test, the vitamin C, wild-type *B. subtilis*, and recombinant *B. subtilis* expressing *GULO* groups exhibited rapid and significant upregulation of CC chemokine mRNA levels in only the spleen at 6 h compared to the control group. However, CC chemokine mRNA expression in the recombinant *B. subtilis* expressing *GULO* group peaked at 12 h in both the liver and spleen, followed by a decline at 48 h across all experimental diet groups (Figure 4).

**Figure 5:**
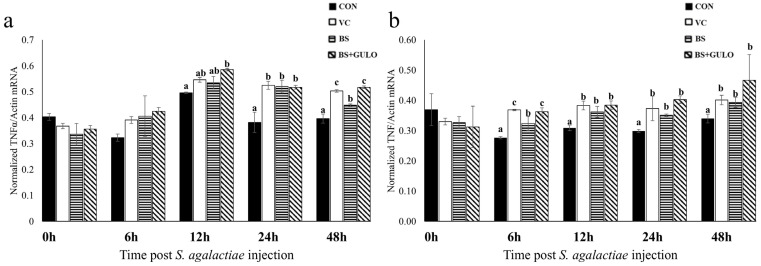
Quantitative real-time PCR analysis of tumor necrosis factor α expression in the spleen (**a**) and liver (**b**) of Nile tilapia in response to *S. agalactiae* at different time points following the 30-day feeding trial (*n* = 3). The different letters on each bar indicate significant differences at *p* < 0.05. Abbreviations: a basal diet (CON); a basal diet + vitamin C (VC); a basal diet + wild-type *B. subtilis* (BS); and a basal diet + recombinant *B. subtilis* (BS+GULO).

In the case of TNFα, significant mRNA upregulation was observed in the vitamin C, wild-type *B. subtilis*, and recombinant *B. subtilis* expressing *GULO* groups at 6 h only in the liver compared to the control group, persisting until 48 h post-injection. In the spleen, significant increases in TNFα mRNA levels were detected at 12 h post-injection only in the recombinant *B. subtilis* expressing *GULO* group compared to the control group, followed by gradual decreases at 24 and 48 h (Figure 5).

## 4. Discussion

Advances in biotechnology have led to novel approaches to alleviate the vulnerability associated with the application of dietary vitamin C supplementation in aquaculture [30,54,55,56,57]. In this study, a recombinant probiotic *B. subtilis* expressing *GULO* was successfully constructed and administered as a dietary supplement to Nile tilapia. To evaluate its potential as a growth promoter and immunostimulant, the expression of L-gulonolactone oxidase produced by probiotic *B. subtilis* was validated through Western blot analysis before being applied as a dietary supplement. After 30 days of the feeding trial, the fish fed a diet supplemented with either wild-type or recombinant probiotic *B. subtilis* expressing *GULO* showed a significantly improved PER, correlating with an increase in fish weight gain. The significant difference may have been due to the presence of the protease-producing capacity of isolated *B. subtilis* to enhance the digestibility of protein content, as reported in our previous study [42].

Interestingly, after 90 days of the feeding trial, the fish fed a diet supplemented with recombinant *B. subtilis* expressing *GULO* showed the highest improvements in final weight, weight gain, specific growth rate, average daily gain, and relative growth rate. This phenomenon may be attributed to the incorporation function of probiotics and vitamin C in enhancing the digestibility and absorption of nutrients within the fish’s body. Numerous studies have reported that dietary supplementation with *B. subtilis* can enhance intestinal digestive enzyme activities, thereby leading to an improvement in the growth performance of the fish [58]. Meanwhile, several pieces of evidence have supported the positive impact of vitamin C on nutrient utilization within metabolic processes and protein synthesis, resulting in a beneficial influence on the growth performance of aquatic animals [59]. Nevertheless, the effect of dietary supplementation with vitamin C can vary based on fish species, age, and size; the form of vitamin C; and differences in experimental conditions, as well as the health status and stress levels of the fish [31,60].

In this study, HPLC and qRT-PCR analyses were conducted to validate and confirm the role of L-gulonolactone oxidase, produced by probiotic *B. subtilis*, in the biosynthesis of vitamin C. This was supported by the significant increase in serum vitamin C of fish fed recombinant *B. subtilis* expressing *GULO* for 90 days compared to the control group. The increase in serum ascorbic acid levels in fish fed recombinant *B. subtilis* expressing *GULO* corresponded to their growth performance results. This result aligns with several previous studies that have documented the advantageous effects of using both *B. subtilis* and vitamin C as supplements in aquafeed, aiming to improve the overall growth of fish [13,22,59,61,62,63].

Beyond their role in enhancing growth performance, vitamin C and probiotics are recognized as immunomodulators that elicit immune responses in fish. According to our previous study [42], the probiotic *B. subtilis* isolated from the intestine of Nile tilapia demonstrates substantial tolerance to the hostile environment of the gastrointestinal (GI) tract, thus increasing its chances of survival and colonization on the internal surfaces of the GI tract. Like other probiotics, the presence of probiotic *B. subtilis* in the GI tract could activate the immune system of Nile tilapia through signaling by toll-like receptors (TLRs) on intestinal epithelial cells and antigen-presenting cells (APCs) [64]. Meanwhile, the concentration of vitamin C in leukocytes and tissues has been reported to stimulate the activity of innate immune responses [65].

In this study, fish fed a diet supplemented with vitamin C-, wild-type *B. subtilis*-, and *B. subtilis* expressing *GULO* showed a significant increase in LZM following 30 and 90 days of the feeding trial. The results of LZM activity confirmed the vital role of probiotic *B. subtilis* and vitamin C in enhancing innate immunity through the mechanism of this enzyme. Similarly, several studies have stated that the supplementation with both vitamin C and probiotic *B. subtilis* in fish diets could stimulate LZM activity by activating myeloid cells (macrophages, monocytes, and neutrophils) [13,66,67]. In fish, LZM has emerged as a powerful innate defense that exerts antimicrobial activity directly against Gram-positive bacteria or indirectly against Gram-negative bacteria after disrupting the bacterial cell wall through the action of complements and other enzymes.

Regarding total Ig, a significant difference was detected only at day 90, suggesting that both vitamin C and probiotics may require prolonged administration to induce a measurable adaptive immune response. The lack of significant differences on day 30 may be attributed to the time needed for these interventions to optimize immunomodulation [68]. Interestingly, total Ig and LZM levels at day 30 were significantly higher than those at day 90 in the groups of fish fed a diet supplemented with vitamin C, *B. subtilis* expressing *GULO*, and the wild-type *B. subtilis*. This result demonstrated the immunostimulatory function of vitamin C and probiotic *B. subtilis* to stimulate the total Ig and LZM in Nile tilapia, which could enhance immune response. This improvement is attributed to factors such as the stabilization of gut microbiota, cumulative probiotic effects, physiological adaptation, and enhanced immunological responses over time [69,70].

In the case of ACH_50_, a significant difference in ACH_50_ levels between day 30 and day 90 of the feeding trial was observed only in the groups of fish fed a diet supplemented with *B. subtilis* expressing *GULO* and the wild-type *B. subtilis*. This finding indicates that the continuous administration of *B. subtilis* could enhance the ACH_50_ activity of Nile tilapia, consistent with evidence from previous studies [6,7]. The continuous administration of probiotics led to an increase in complement component 3 (C3) through the stimulation of cytokines following recognition by TLRs, as described above [71,72]. Moreover, C3 is a central component in three complement pathways (classical, alternative, and lectin pathway). It interacts with other proteins in the complement cascade to form the membrane attack complex (MAC), ultimately killing pathogens. In addition, previous studies have demonstrated that supplementation with an appropriate amount of vitamin C can enhance complement activity in fish [25,73,74].

In addition to the function described above, LZM and complement components (C1q, C3b, and Bb) also act as an innate opsonin that binds bacteria to accelerate and facilitate phagocytic activity in fish. This is evident in our phagocytic activity results, where fish fed with dietary supplementation of vitamin C and recombinant probiotic *B. subtilis* expressing *GULO* exhibited significantly higher phagocytic activity. In general, phagocytes generate reactive oxygen species (ROS) as a key component of their pathogen-killing mechanism. Consequently, an enhanced antioxidant system protects these cells from self-inflicted oxidative damage, thereby maintaining their effectiveness in eliminating infections.

In teleosts, SOD, MDA, GSH-Px, and CAT are the main antioxidant enzymes that protect fish from oxidative stress damage caused by free radicals. In this study, dietary supplementation with vitamin C and recombinant *B. subtilis* expressing *GULO* led to higher contents of TAC, SOD, CAT, and GSH-Px and lower levels of MDA in the serum of Nile tilapia compared to control groups. In the BS group, a significant decrease was only observed in MDA levels, indicating decreased lipid peroxidation and a reduction in oxidative damage to cellular membranes [75]. These findings could primarily be attributed to the supplementation with vitamin C in the fish diet rather than probiotics. The enhancement of antioxidant enzymes possibly occurs because of vitamin C’s ability to readily donate electrons, aligning with previous findings in several teleost species [22,49,67,76]. In an intensive culture system, Nile tilapia frequently encounters periods of stress at any time. The stress condition can cause an imbalance between reactive oxygen species (ROS) and endogenous antioxidants in cells and tissues, potentially leading to cell and tissue damage. Hence, the continuous supply of exogenous antioxidants, such as vitamin C supplementation in fish diets, is necessary to counteract the adverse effects of oxidative stress.

In Thailand, *S. agalactiae* has emerged as a major pathogenic bacterium, causing severe economic losses in tilapia farming [77]. To investigate the effect of dietary supplementation with recombinant probiotic *B. subtilis* expressing *GULO* on immune response following a challenge with *S. agalactiae*, Nile tilapia were intraperitoneally injected with this bacterium after a 30-day feeding trial. The results showed that the ACH_50_ level rapidly increased at 6 h post-injection in fish fed vitamin C and recombinant probiotics compared to the control group. Meanwhile, total Ig levels were subsequently elevated at 24 and 48 h post-injection in the same groups.

The rapid increase in ACH_50_ indicates its ability to attenuate/limit the spread of invading pathogens, a consequence of activation by either recombinant probiotic *B. subtilis* or vitamin C. The elevation of total Ig at 24 and 48 h post-injection could result from the opsonization facilitated by immune genes such as cytokines, phagocytes, and complement components, leading to the activation of the phase of adaptive immune responses. In addition, a significant increase in total Ig levels after the injection of *S. agalactiae* suggests a more robust humoral immune response, with increased production of antibodies that play a vital role in pathogen recognition and neutralization [78]. In the challenge test, LZM showed a significant elevation in levels at all time points in fish fed with vitamin C, wild-type, and recombinant probiotics compared to the control group. These results reflect the enhanced ability of lysozyme, due to vitamin C and probiotic *B. subtilis*, to eliminate *S. agalactiae* in Nile tilapia. Probiotic *B. subtilis* is recognized for its role in regulating the fish gut’s immune response, while vitamin C is notable for reinforcing the immune response and disease resistance, probably attributable to its antioxidant and immunostimulatory properties [4,79].

Under normal conditions, the continuous application of probiotic *B. subtilis* in fish feed influences the TLR4 triggering, which serves as the pattern-recognition receptor that initiates the activation of the immune cascade. Additionally, dietary supplementation with vitamin C not only modulates the production of fish immune cells, contributing to maintaining immune homeostasis, but also plays a role in disease resistance by activating the expression of inflammatory cytokines under stress conditions [80]. In the challenge test, mRNA levels of pro-inflammatory cytokines, including CC chemokine and TNFα, in response to *S. agalactiae*, were analyzed among the experimental fish after a 30-day feeding trial using qRT-PCR.

The results indicate a significant and rapid increase in CC chemokine mRNA expression at 6 h post-*S. agalactiae* injection in both the liver and spleen of fish fed diets supplemented with vitamin C and recombinant probiotic *B. subtilis* expressing *GULO*, compared to the control group. A similar pattern was observed for TNFα in the liver of fish fed dietary supplementation with vitamin C, wild-type *B. subtilis*, and recombinant probiotic *B. subtilis* expressing *GULO*. These findings suggest that both vitamin C and probiotic *B. subtilis* may potentially contribute to enhancing the production and chemoattractant activity of CC chemokine and TNFα.

Moreover, only the recombinant probiotic *B. subtilis* expressing *GULO* group exhibited a significant increase in TNFα expression in the spleen at 12 h. The rapid upregulation of inflammatory gene expression facilitated the recruitment of white blood cells to the site of infection during the initial stage [53]. Furthermore, our previous in vitro study confirmed that the potential probiotic *B. subtilis* used in this study exhibits antibacterial activity and effectively antagonizes pathogenic *S. agalactiae* [34]. Together, these findings suggest that the enhanced antagonistic activity against *S. agalactiae* in recombinant *B. subtilis* may result from the combined effect of *B. subtilis* and vitamin C, modulating both innate and adaptive immunity in Nile tilapia.

## 5. Conclusions

In conclusion, based on the overall results, dietary supplementation with recombinant probiotic *B. subtilis* expressing *GULO* could be considered for prophylactic and therapeutic applications, owing to the combined effects of vitamin C and probiotic *B. subtilis*. In prophylactic roles, supplementation with recombinant *B. subtilis* expressing *GULO* in normal fish resulted in improvements in growth performance, antioxidant activity, and immune responses. In addition, it may demonstrate therapeutic potential in the early stages of *S. agalactiae* infections due to its ability to enhance immune responses and pro-inflammatory cytokine production and to exhibit antagonistic properties against *S. agalactiae*. These could help to reduce the prevalence of the disease in Nile tilapia, particularly in the intensive aquaculture industry, which often relies on the application of drugs and chemicals. However, this study was primarily conducted under strictly controlled laboratory conditions. Further research is needed to assess long-term effects, optimize the dosage under field conditions, and improve signal peptides for enhanced recombinant *B. subtilis* protein expression.

## Figures and Tables

**Table 1:** The list of oligonucleotide sequences used in this study.

Primer Name	5′ to 3′ Nucleotide Sequences	Size (bp)	Ta (°C)	Purposes	Accession No.
H-B-*GULO*F	AAGCTTGGATCCATGGTTCACGGCCAAGGAGG	1323	55	Cloning	XM_015285218
H-X-*GULO*R	AAGCTTCTCGAGGTAGAACACCTTTTCCAGAT			Cloning	
*GULO*-qPCRF	ACAGGGACGCACAACACTGG	172	59	qRT-PCR	XM_015285218
*GULO*-qPCRR	TGACGGTGAGCACAACACCC			qRT-PCR	
β-actinF	ACAGGATGCAGAAGGAGATCACAG	155	55	qRT-PCR	KJ126772.1
β-actinR	GTACTCCTGCTTGCTGATCCACAT			qRT-PCR	
*On*CC-F	ACAGAGCCGATCTTGGGTTACTTG	229	55	qRT-PCR	KJ535436.1
*On*CC-R	TGAAGGAGAGGCGGTGGATGTTAT			qRT-PCR	
*On*TNF-αF	GAGGCCAATAAAATCATCATCCC	161	55	qRT-PCR	NM_001279533
*On*TNF-αR	CTTCCCATAGACTCTGAGTAGCG			qRT-PCR	

Ta = Annealing temperature.

**Table 2:** Growth performance of Nile tilapia fed experimental diets for 30 and 90 days post-feeding.

Diet	Initial Weight	Final Weight	Initial Length	Final Length	Weight Gain	FCR	ADG	SGR	RGR	PER
	(g)	(g)	(cm)	(cm)	(g)		(g day^−1^)	(% day^−1^)	(%)	
30 days										
CON	75.11 ± 5.81	141.47 ± 13.78	15.97 ± 0.58	19.40 ± 0.51	66.36 ± 8.78	1.52 ± 0.06	2.21 ± 0.29	0.91 ± 0.06	88.23 ± 7.43	2.14 ± 0.07 ^a^
VC	73.16 ± 2.39	138.80 ± 6.24	15.71 ± 0.19	19.19 ± 0.25	65.64 ± 4.90	1.55 ± 0.02	2.19 ± 0.16	0.93 ± 0.05	89.74 ± 6.13	2.15 ± 0.02 ^a^
BS	80.18 ± 1.34	151.29 ± 10.30	16.30 ± 0.13	19.73 ± 0.46	71.11 ± 10.24	1.44 ± 0.07	2.57 ± 0.07	0.97 ± 0.04	95.94 ± 4.78	2.32 ± 0.12 ^b^
BS + GULO	78.56 ± 3.50	158.64 ± 3.55	16.21 ± 0.45	19.99 ± 0.09	80.09 ± 0.50	1.41± 0.01	2.67 ± 0.02	1.02 ± 0.03	102.09 ± 4.56	2.37 ± 0.02 ^b^
90 days										
CON	75.11 ± 5.81	268.75 ± 7.19 ^a^	15.97 ± 0.58	25.15 ± 1.74	195.72± 0.73 ^a^	1.60 ± 0.07 ^b^	2.17 ± 0.01 ^a^	0.63 ± 0.03 ^a^	268.99 ± 22.79 ^a^	2.23 ± 0.09
VC	73.16 ± 2.39	312.17 ± 13.12 ^b^	15.71 ± 0.19	25.83 ± 0.35	237.73 ± 14.47 ^b^	1.52 ± 0.09 ^ab^	2.64 ± 0.16 ^b^	0.69 ± 0.03 ^a^	319.60 ± 24.91 ^a^	2.20 ± 0.13
BS	80.18 ± 1.34	327.06 ± 12.45 ^b^	16.30 ± 0.13	25.94 ± 0.49	246.88 ± 12.08 ^b^	1.54 ± 0.03 ^ab^	2.74 ± 0.13 ^b^	0.68 ± 0.02 ^a^	307.93 ± 14.78 ^a^	2.17 ± 0.05
BS + GULO	78.56 ± 3.50	381.25 ± 8.13 ^c^	16.21 ± 0.45	27.46 ± 0.41	302.75 ± 3.18 ^c^	1.42 ± 0.00 ^a^	3.36 ± 0.04 ^c^	0.76 ± 0.02 ^b^	386.31 ± 20.30 ^b^	2.34 ± 0.00

Means with a different superscript in each column differed significantly (*p* < 0.05). Values are means ± SD of ten replicates. Abbreviations: a basal diet (CON); a basal diet + vitamin C (VC); a basal diet + wild-type *B. subtilis* (BS); and a basal diet + recombinant *B. subtilis* (BS+GULO).

**Table 3:** Accumulation of serum vitamin C in Nile tilapia fed with experimental diets for 90 days.

Diet	Ascorbic Acid Level (µg mL^−1^)
CON	5.88 ± 1.21 ^a^
VC	20.29 ± 2.91 ^c^
BS	5.92 ± 0.66 ^a^
BS+GULO	10.43 ± 1.20 ^b^

Significant differences among diet groups are denoted by different letters (*p* < 0.05). Values are means ± SD of three replicates. Abbreviations: a basal diet (CON); a basal diet + vitamin C (VC); a basal diet + wild-type *B. subtilis* (BS); and a basal diet + recombinant *B. subtilis* (BS+GULO).

**Table 4:** Antioxidant parameters of Nile tilapia fed experimental diets for 90 days.

Diet	TAC	SOD	MDA	GSH-Px	CAT
	µmol mL^−1^	U mL^−1^	nmol mL^−1^	U mL^−1^	nmol min^−1^ mL^−1^
CON	28.16 ± 0.91 ^a^	3.32 ± 0.10 ^a^	0.36 ± 0.006 ^c^	0.068 ± 0.001 ^a^	10.39 ± 0.83 ^a^
VC	38.85 ± 1.32 ^b^	4.34 ± 0.34 ^b^	0.23 ± 0.025 ^a^	0.121 ± 0.013 ^b^	31.27 ± 2.59 ^c^
BS	32.03 ± 1.02 ^ab^	4.04 ± 0.12 ^ab^	0.31 ± 0.002 ^b^	0.088 ± 0.007 ^ab^	17.29 ± 1.32 ^ab^
BS+GULO	36.01 ± 1.78 ^b^	4.69 ± 0.32 ^b^	0.28 ± 0.006 ^b^	0.117 ± 0.016 ^b^	20.34 ± 4.16 ^b^

Means with a different superscript in each column differed significantly from each other (*p* < 0.05). Values are means ± SD of three replicates. Abbreviations: a basal diet (CON); a basal diet + vitamin C (VC); a basal diet + wild-type *B. subtilis* (BS); and a basal diet + recombinant *B. subtilis* (BS+GULO).

## Data Availability

The data that support the findings of this study are available on request from the corresponding author.

## Supplementary Materials

The following supporting information can be downloaded at: https://www.mdpi.com/article/10.3390/antiox14010050/s1, Figure S1: Sequence alignment of *G. gallus GULO* cDNA after cloning into the pBES expression vector; Figure S2: Construction of probiotic *B. subtilis* expressing *GULO*; Figure S3: Western blot analysis of pBES*GULO*; Figure S4: Melting curve analysis of quantitative real-time PCR; Figure S5: HPLC chromatograms of ascorbic acid in Nile tilapia serum; Figure S6: Multiple alignments of the GULO amino acid sequence with known eukaryotic GULO protein homologs.
