# Supplementary material for: Enhancement of Growth, Antioxidant Activity, and Immunity in Nile Tilapia (Oreochromis niloticus) Through Recombinant Bacillus subtilis Expressing L-Gulonolactone Oxidase
Source: Antioxidants (Basel). 2025 Jan 4;14(1):50. doi: 10.3390/antiox14010050 (PMC11759777; doi:10.3390/antiox14010050)
Supplement: Supplementary file 1 [file antioxidants-14-00050-s001.zip › antioxidants-3383021-supplementary.pdf]

Supplementary data

Enhancement of Growth, Antioxidant Activity, and Immunity in Nile Tilapia (*Oreochromis niloticus*) Through Recombinant *Bacillus subtilis* Expressing L-Gulonolactone Oxidase

Jirawadee Kaewda, Surintorn Boonanuntanasarn, Papungkorn Sangsawad, Pimpisut Manassila and Chatsirin Nakharuthai *

School of Animal Technology and Innovation, Institute of Agricultural Technology, Suranaree University of Technology, Nakhon Ratchasima 30000, Thailand

*Corresponding author:

Email: chatsirin_nak@sut.ac.th (Chatsirin Nakharuthai)

a

```
                                     Forward primer
                                 5'-AAGCTTGGATCCATGGTTCACGGCCAAGGAGG-3'
GULO+pBES1__sequence      AAGCTTGGATCCATGGTTCACGGCCAAGGAGGATTCAAGTTCCAGAACTGGGCCAAGACG
XM_015285218.1            ------------ATGGTTCACGGCCAAGGAGGATTCAAGTTCCAGAACTGGGCCAAGACG
                                      ************************************************

GULO+pBES1__sequence      TATGGCTCTTCCCCAGAGCTGTACTTCCAGCCCACCTCAGTGGAGGAGATCCGGGAGATC
XM_015285218.1            TATGGCTCTTCCCCAGAGCTGTACTTCCAGCCCACCTCAGTGGAGGAGATCCGGGAGATC
                          ************************************************************

GULO+pBES1__sequence      CTGGATATGGCCCGGCAGAGGAACAAGAGGGTGAAGGTGGTGGGGGGCGGCCACTCGCCC
XM_015285218.1            CTGGATATGGCCCGGCAGAGGAACAAGAGGGTGAAGGTGGTGGGGGGCGGCCACTCGCCC
                          ************************************************************

GULO+pBES1__sequence      TCTGACATCGCCTGCACTGATGACTTCATGATCCAGATGGGGAAGATGAACAAGGTCCTC
XM_015285218.1            TCTGACATCGCCTGCACTGATGACTTCATGATCCAGATGGGGAAGATGAACAAGGTCCTC
                          ************************************************************

GULO+pBES1__sequence      AAGGTGGACAAGGAGAAGCAGCAGGTGACGGTGGAAGGTGGGATCTTCCTCTCGGATCTG
XM_015285218.1            AAGGTGGACAAGGAGAAGCAGCAGGTGACGGTGGAAGGTGGGATCTTCCTCTCGGATCTG
                          ************************************************************

GULO+pBES1__sequence      GACGTGGAGCTGAGCAAGCACGGGCTGGCACTGGCCAACTTAGGAGCCGTTTCTGAGGTG
XM_015285218.1            AACGTGGAGCTGAGCAAGCACGGGCTGGCACTGGCCAACTTAGGAGCCGTTTCTGAGGTG
                          .***********************************************************

GULO+pBES1__sequence      GCAGCAGCTGGTGTGATTGGGACAGGGACGCACAACACTGGGATCAAGCATGGCATCCTC
XM_015285218.1            GCAGCAGCTGGTGTGATTGGGACAGGGACGCACAACACTGGGATCAAGCATGGCATCCTC
                          ************************************************************

GULO+pBES1__sequence      CCCACCCAGGTTGTAGGGCTCTCACTGCTGACAGCCTCAGGGGACATCCTGGAGTGCTCC
XM_015285218.1            CCCACCCAGGTTGTAGGGCTCTCACTGCTGACAGCCTCAGGGGACATCCTGGAGTGCTCC
                          ************************************************************

GULO+pBES1__sequence      GAGTCCATCAATGCAGATATCTTCCAGGCTGCCCGCCTGCACCTTGGCTGCCTGGGTGTT
XM_015285218.1            GAGTCCATCAATGCAGATATCTTCCAGGCTGCCCGCCTGCACCTTGGCTGCCTGGGTGTT
                          ************************************************************

GULO+pBES1__sequence      GTGCTCACCGTCACCTTCCAGTGCGTGCCCCAGTTCCACCTGCATGAGGTGACCTTTCCA
XM_015285218.1            GTGCTCACCGTCACCTTCCAGTGCGTGCCCCAGTTCCACCTGCACGAGGTGACCTTTCCA
                          ******************************************** ***************

GULO+pBES1__sequence      TCCACCCTCACTGAGGTCCTCAATCACCTTGATGACCACCTAAAGAGATCCCAATACTTC
XM_015285218.1            TCCACCCTCACTGAGGTCCTCAATCACCTTGATGACCACCTAAAGAGATCCCAATACTTC
                          ************************************************************

GULO+pBES1__sequence      CGATTCCTGTGGTTTCCACACAGTGAGAACGTCACTGTCATCTACCAGGACCCCACCAAC
XM_015285218.1            CGATTCCTGTGGTTCCCACACAGTGAGAACGTCACTGTCATCTACCAGGACCCCACCAAC
                          ************** *********************************************

GULO+pBES1__sequence      AAGCCCCCCTCTTCCTCCGCTAACTGGTTTTGGGATTATGCTGTTGGGTACTACTTGCTG
XM_015285218.1            AAGCCCCCCTCTTCCTCCGCTAACTGGTTTTGGGATTATGCTGTTGGGTACTACTTGCTG
                          ************************************************************

GULO+pBES1__sequence      GAGTTTCTCCTCTGGATCAGCACCTTCGTGCCCAGCTTGGTGTGCTGGATCAACCGCTTC
XM_015285218.1            GAGTTTCTCCTCTGGATCAGCACCTTCGTGCCCAGCTTGGTGTGCTGGATCAACCGCTTC
                          ************************************************************

GULO+pBES1__sequence      TTCTTCTGGCTCCTCTTCAGCTCCCGGGTGGAGAACATCAATGTCAGCTACAAGATCTTC
XM_015285218.1            TTCTTCTGGCTTCTCTTCAGCTCCCGGGTGGAGAACATCAATGTCAGCTACAAGATCTTC
                          *********** ************************************************

GULO+pBES1__sequence      AACTACGAGTGTCGCTTCAAGCAGCATGTGCAAGACTGGGCCATTCCCATTGAGAAGACA
XM_015285218.1            AACTACGAGTGCCGCTTCAAGCAGCATGTGCAAGACTGGGCCATCCCCATTGAGAAGACA
                          *********** ******************************** ***************

GULO+pBES1__sequence      AAGGAAGCACTGCTGGAGCTGAAGGCTGCCCTGGAGAACAACCCCAAGATGGTGGCCCAC
XM_015285218.1            AAGGAAGCACTGCTGGAGCTGAAGGCTGCCCTGGAGAACAACCCCAAGATGGTGGCCCAC
                          ************************************************************

GULO+pBES1__sequence      TACCCTGTGGAGGTGCGCTTTGCTCGAGCGGATGAGATCTGGCTGAGCCCCTGCTTCCAG
XM_015285218.1            TACCCTGTGGAGGTGCGCTTTGCTCGAGCGGATGAGATCTGGCTGAGCCCCTGCTTCCAG
                          ************************************************************

GULO+pBES1__sequence      AGGGACAGCTGCTACATGAACATCATCATGTACAGGCCCTATGGGAAGAACGTGCCCCGG
XM_015285218.1            AGGGACAGCTGCTACATGAACATCATCATGTACAGGCCCTATGGGAAGAACGTGCCCCGG
                          ************************************************************

GULO+pBES1__sequence      CTCAACTACTGGCTGACCTACGAGGGCATCATGAAGAAGTATGGTGGGAGACCACACTGG
XM_015285218.1            CTCAACTACTGGCTGACCTACGAGGGCATCATGAAGAAGTATGGTGGGAGACCACACTGG
                          ************************************************************

GULO+pBES1__sequence      GCAAAGGCCCACAGCTGCACCCGCAAGGATTTTGAGAAGATGTATCCGGCCTTCCCCAAA
XM_015285218.1            GCAAAGGCCCACAGCTGCACCCGCAAGGATTTTGAGAAGATGTATCCGGCCTTCCCCAAA
                          ************************************************************

GULO+pBES1__sequence      TTCTGCTCCGTCCGGGATAAGCTGGACCCTACAGGGATGTTCCTGAACACCTATCTGGAA
XM_015285218.1            TTCTGCTCCGTCCGGGATAAGCTGGACCCTACAGGGATGTTCCTGAACACCTATCTGGAA
                          ************************************************************
                                                                             3-'TGGAA

GULO+pBES1__sequence      AAGGTGTTCTACCTCGAGAAGCTT
XM_015285218.1            AAGGTGTTCTACTGA------------
                          ************
                         AAGGTGTTCTACCTCGAGAAGCTT-5'
                                      Reverse primer
```

b

5'3' Frame 1

```
MVHGQGGFKFQNWAKTYGSSPELYFQPTSVEEIREILDMARQRNKRVKVVGGGHSPSDIACTDDFMIQMGKMNKVLKVDKEKQQVTVEGGIFLSDLDVE
LSKHGLALANLGAVSEVAAAGVIGTGTHNTGIKHGILPTQVVGLSLLTASGDILECSESINADIFQAARLHLGCLGVVLTVTFQCVPQFHLHEVTFPST
LTEVLNHLDDHLKRSQYFRFLWFPHSENVTVIYQDPTNKPPSSSANWFWDYAVGYYLLEFLLWISTFVPSLVCWINRFFFWLLFSSRVENINVSYKIFN
YECRFKQHVQDWAIPIEKTKEALLELKAALENNPKMVAHYPVEVRFARADEIWLSPCFQRDSCYMNIIMYRPYGKNVPRLNYWLTYEGIMKKYGGRPHW
AKAHSCTRKDFEKMYPAFPKFCSVRDKLDPTGMFLNTYLEKVFY-
```

Theoretical p*I*/Mw: 7.96 / 50497.19 (https://web.expasy.org/translate/)

Figure S1. Sequence alignment of *G. gallus GULO* cDNA after cloning into the pBES expression vector and the full-length *GULO* cDNA of *G. gallus* (accession no. XM_015285218) published on the GenBank database (a). Structural analyses of the amino acid sequences of *GULO* protein in *G. gallus* revealed that the whole protein had a molecular weight and an isoelectric point (*p*I) of 50.5 kDa and 7.96, respectively (b).

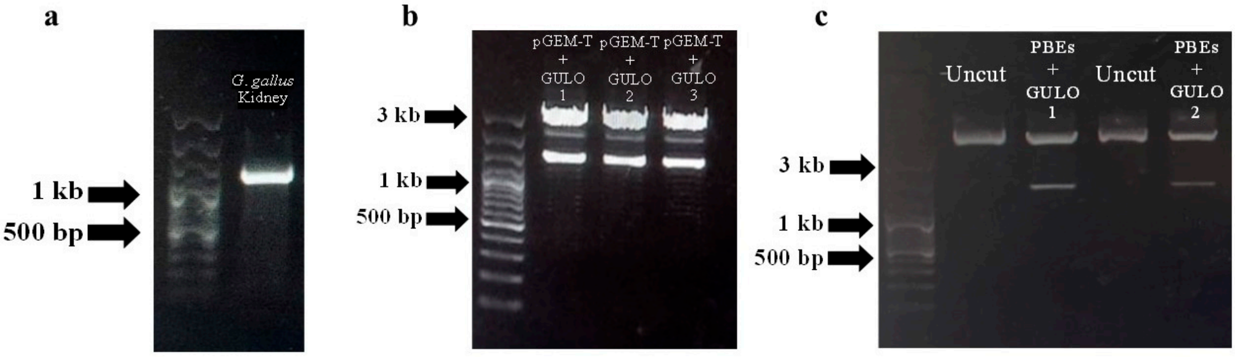

Figure S2. Construction of probiotic *B. subtilis* expressing *GULO*. Amplification of the full-length *GULO* cDNA from *G. gallus* using semi-quantitative RT-PCR (a); Double restriction enzyme digestion result after cloning into the pGEM-T vector; 3 Kb of linearized pGEM-T and 1.2 Kb of target gene with digested with *Bam*HI and *Hind*III (b). Double restriction enzyme digestion result after cloning into the pBES expression vector; 5 Kb of linearized pBES and 1.2 Kb of target gene with digested with *Bam*HI and *Hind*III and compared to undigested pBES plasmid (c).

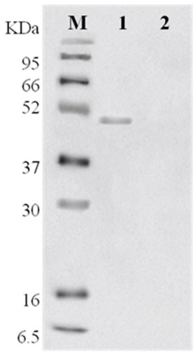

Figure S3. Western blot analysis of pBES*GULO* shows protein bands of *GULO* at a molecular weight of 50 kDa. Lane 1: protein marker, 2: pBES*GULO*, and 3: wild-type *B. subtilis* (negative control).

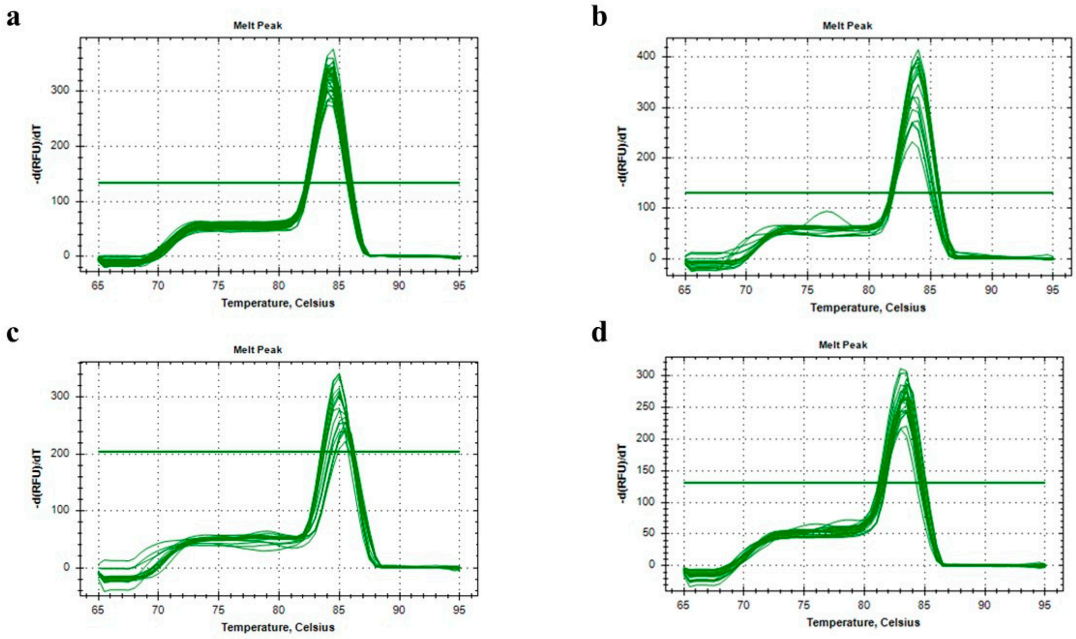

Figure S4. Quantitative real-time PCR. Melting curve analysis reveals melting peaks of β-actin at 83.5°C (a), TNFα at 83.5°C (b), *GULO* at 85.5°C (c), and CC chemokine at 83.5°C (d).

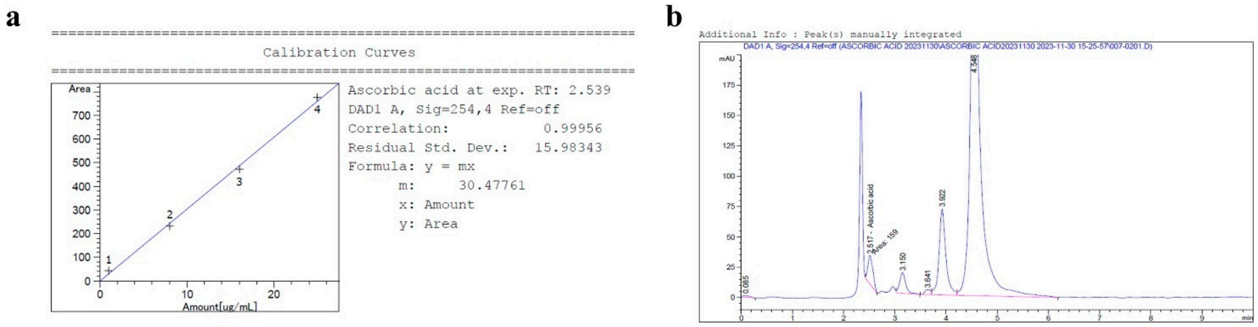

Figure S5. HPLC chromatograms of ascorbic acid in Nile tilapia serum; Calibration curve ($R^2 = 0.99$) (a) and BS+GULO group (b).

The N-terminal FAD-binding domain

```
                                                                        X[G/R]x[G/S][H/L/K][S/G]
Acipenser ruthenus                --MVQGMEGFQFQNWALTYSSNPELYFQPTSIEEVRQILELARQRGKKVKVVGGGHSPSD
Polyodon spathula                 LKMVQGMEGYHFQNWALTYSSNPELYFQPTSIEEVRQILELARQRSKKVKVVGGGHSPSD
Lepisosteus oculatus              --MIQGTEGYHFQNWAQTYSCHPEQYFQPSSVEDVRQILELARRCSKRVKVVGGGHSPSD
Amia calva                        --MVQGKAGYRFQNWAQTYSSCPEKYFQPSSVEDVRQILAMARQSSKRVKVVGGGHSPSD
Callorhinus ursinus               --MVHGYKGVKFQNWAKTYGCCPEIYYQPTSVEEVREVLALARQQNKRVKVVGGGHSPSD
Canis lupus familiaris            --MVHGYKGVKFQNWARTYGCCPEMYFQPTSVEEVREVLALARQQNKRVKVVGGGHSPSD
Felis catus                       --MVHGYKGVKFQNWARTYGCCPEMYYQPTSVEEVREVLALARQQNKRVKVVGGGHSPSD
Mus musculus                      --MVHGYKGVQFQNWAKTYGCSPEMYYQPTSVGEVREVLALARQQNKKVKVVGGGHSPSD
Mauremys reevesii                 --MVHGQGGFKFQNWARTYGSSPELYFQPTSVEEIKEILDLARQRSKRVKVVGGGHSPSD
Chelonoidis abingdonii            --MVHGQGGFKFQNWARSYGSSPELYFQPTSVEEIKEILDLARQRSKRVKVVGGGHSPSD
Alligator mississippiensis        --MVHGHGGVQFQNWAKTYGSSPELYFQPTSVEEIKEILELARQRNKRVKVVGGGHSPSD
Gallus gallus                     --MVHGQGGFKFQNWAKTYGSSPELYFQPTSVEEIREILDMARQRNKRVKVVGGGHSPSD
Meleagris gallopavo               SSMVHGQGGVKFQNWAKTYGSSPELYFQPTSVEEIREILDMARQRNKRVKVVGGGHSPSD
Aquila chrysaetos                 --MVHGQGGVKFQNWAKTYGSSPELYFQPTSVEEIREILDMARQRDKRVKVVGGGHSPSD
Haliaeetus albicilla              GHEVHGQGGFKFQNWAKTYGSSPELYFQPTSVEEIREILDMARQRDKRVKVVGGGHSPSD
Opisthocomus hoazin               LVQVHGQGGVKFQNWAKTYGSSPELYFQPTSVEEIREILDMARQRDKRVKVVGGGHSPSD
Scyliorhinus torazame             --MDQGTMGYQFENWATTYSCEPELYFEPTTVEEIRQILELANQRNKRVKVVGCGHSPSD
                                      :*  * :*:*** :*.. ** :::*:.  ::::* :*.: .*:***** ******
```

```
Acipenser ruthenus                IACTEDFMIRMDKMNKVLEVDKKKRQATVEAGIFLCDLNEELARHGLAMSNLGAVAEVAA
Polyodon spathula                 IACTEDFMIRMDKMNKVLEVDKEKRQATVEAGIFLCDLNEELARHGLAMSNLGAVAEVAA
Lepisosteus oculatus              IACTDDFMMRMDKMNKVLEVDEEKRQVTVEAGIFLCDLNEELAKHGLALSNLGAVAEVSA
Amia calva                        IACTDEFMISLDKMNRVLEVNKEERRVTVEAGIFLCDLNEELTKHGLALSNLGAVAEVAA
Callorhinus ursinus               IACTDGFMIHMGKMNRVLQVDTEKKQVTVEAGILLADLHPQLGKHGLALSNLGAVSDVTA
Canis lupus familiaris            IACTDGFMIHMGKMNRVLQVDTEKKQVTVEAGILLADLHPQLGKHSLALSNLGAVSDVTA
Felis catus                       IACTDGFMIHMGKMNRVLQVDTEKKQVTVEAGILLSDLHPQLGKHGLALSNLGAVSDVTA
Mus musculus                      IACTDGFMIHMGKMNRVLQVDKEKKQVTVEAGILLTDLHPQLDKHGLALSNLGAVSDVTV
Mauremys reevesii                 IACTDDFMVQMGKMNKILKVDEEKRQVMVEAGILLSDLNIELSKYGLALPNLGAVSEVAA
Chelonoidis abingdonii            IACTDDFMVQMGKMNKILKVDKEKRQVMVEAGILLSDLNVELSKYGLALPNLGAVSEVAA
Alligator mississippiensis        IACTDGFMIQMGKMNKILKVDKEKRQATVEAGILLSDLNVELTKYGLALANLGAVSEVAA
Gallus gallus                     IACTDDFMIQMGKMNKVLKVDKEKQQVTVEGGIFLSDLNVELSKHGLALANLGAVSEVAA
Meleagris gallopavo               IACTDDFMIQMGKMNKVLKVDKEKQQVTVEGGIFLSDLNVELSKHGLALANLGAVSEVAA
Aquila chrysaetos                 IACTDDFMIQMGKMNRVLKVDKEKQQVTVEGGIFLSDLNVELSKHGLALANLGAVSEVAA
Haliaeetus albicilla              IACTDDFMIQMGKMNRVLKVDKEKQQVTVEGGIFLSDLNIELSKHGLALANLGAVSEVAA
Opisthocomus hoazin               IACTDDFMIQMGKMNRVLKVDKEKQQVTVEGGIFLSDLNVELSKHGLALANLGAVSEVAA
Scyliorhinus torazame             IACTDNYLVRLNKLNRILQVDKERKWITAEAGILLSDLNEKLDALGLALSNIGAVSDVAL
                                  ****  ::: :.*:*::*:*: :.:   .*.**:* **: :*   .**:.*:***.:*:
```

```
Acipenser ruthenus              AGVIGTGTHNTGIEHGILPTQVVALTLMTAGGEILKCSDSLNEEIFQAARLHLGSLGVIL
Polyodon spathula               AGVIGTGTHNTGIEHGILPTQVVALTLMTAGGEILKCSDSLNEEIFQAARLHLGSLGVIL
Lepisosteus oculatus            AGVTGTGTHNTGVDHGILSTQVVRLSLMTASGEVLHCSESVNEDIFQAARLHLGCLGVII
Amia calva                      AGVIGTGTHNTGIDHGILPTQVVSLSLMTAVGEVLHCSDSVNQDIFQAARLHLGSLGVIL
Callorhinus ursinus             GGVIGSGTHNTGIKHGILATQVVALTLLTADGTILECSESSNAEVFQAARVHLGCLGVIL
Canis lupus familiaris          GGVIGSGTHNTGIKHGILATQVVALTLLTADGTILECSESSNADVFQAARVHLGCLGVVL
Felis catus                     GGVIGSGTHNTGIKHGILPTLVVALTLLTADGTILECSESSNAEVFQAARVHLGCLGVVL
Mus musculus                    GGVIGSGTHNTGIKHGILATQVVALTLMKADGTVLECSESSNADVFQAARVHLGCLGVIL
Mauremys reevesii               AGVIGTGTHNTGIKHGILPTQVVALTLLTASGEILECSESINAEIFQAARLHLGCLGVVL
Chelonoidis abingdonii          AGVIGTGTHNTGIKHGILPTQVVALTLLTASGEVLECSESVNAEIFQAARLHLGCLGVVL
Alligator mississippiensis      AGVIGTGTHNTGIKHGILPTQVVALTLLTAAGEILECSEGSHADIFQAARVHLGCLGVVL
Gallus gallus                   AGVIGTGTHNTGIKHGILPTQVVGLSLLTASGDILECSESINADIFQAARLHLGCLGVVL
Meleagris gallopavo             AGVIGTGTHNTGIKHGILPTQVVGLSLLTASGDILECSESINADIFQAARLHLGCLGVVL
Aquila chrysaetos               AGVIGTGTHNTGIKHGILPTQVVALTLLTASGEILECSESVNADIFQAARLHLGCLGVVL
Haliaeetus albicilla            AGVIGTGTHNTGIKHGILPTQVVALTLLTASGEILECSESINADIFQAARLHLGCLGVVL
Opisthocomus hoazin             AGVIGTGTHNTGIKHGILPTQVVALTLLTASGEILECSESINADIFQAARLHLGCLGVVL
Scyliorhinus torazame           GGVIGTGTHNTGIQHGILATQIVAMTLMTAAGDTLECSNTVNREIFQATRLHLGSLGVVL
                                .*: *:******:.****.* :* ::*:.* *  * **.  : ::***:*:***.***::
```

```
Acipenser ruthenus              DLTIQCVPAFRLLELQFPSTLTEVLDNLDFHMKKSEYFRFLWFPHTENVRVIYQDRTDKP
Polyodon spathula               SLTIQCVPAFRLHELQFPSTLTEVLDNLDFHMKKSEYFRFLWFPHTENVRVIYQDRTDKP
Lepisosteus oculatus            TITFQCQPAFRLHERQFPSTLTEVLDNLDFHRKKSEYFRFLWFPNTEHVRIIYQDRTEKP
Amia calva                      TLTYQCQPAFRLHERQFPSTLTEVLDNLDFHMKKSEYFRFLWFPHTENVRVIYQDRTDKP
Callorhinus ursinus             TITLQCVPQFHLQEISFPSTLEEVLNNLDSHLKKSEYFRFLWFPHSENVSIIYQDHTNKP
Canis lupus familiaris          TVTLQCVPQFHLQEISFPSTLEEVLNNLDGHLKKSEYFRFLWFPHSENVSVIYQDHTNKP
Felis catus                     TITLQCVPQFHLQEISFPSTLDEVLDNLDSHLKKSEYFRFLWFPHSENVSVIYQDHTNKP
Mus musculus                    TVTLQCVPQFHLLETSFPSTLKEVLDNLDSHLKKSEYFRFLWFPHSENVSIIYQDHTNKE
Mauremys reevesii               TITFQCVPEFYLLETTFPSTLQEVLDNLDSHLRRSEYFRFLWFPHSENVSIIYQDHTNKP
Chelonoidis abingdonii          TITFQCVPEFYLLETTFPSTLQEVLDNLDSHLRRSEYFRFLWFPHSENVSVIYQDHTNKP
Alligator mississippiensis      TITFQCVPEFYLQETTFPSTLKEVLDNLDSHLQRSEYFRFLWFPHSENVSVIYQDHTSKP
Gallus gallus                   TVTFQCVPQFHLHEVTFPSTLTEVLNHLDDHLKRSQYFRFLWFPHSENVTVIYQDPTNKP
Meleagris gallopavo             TVTFQCVPQFHLHEVTFPSTLTEVLNHLDDHLKRSQYFRFLWFPHSENVTVIYQDPTNKP
Aquila chrysaetos               TVTFQCVPQFHLHEVAFPSTLTEVLDHLDDHLQRSQYFRFLWFPHSENVSVIYQDPTNKP
Haliaeetus albicilla            TVTFQCVPQFHLHEVAFPSTLTEVLDHLNDHLQRSQYFRFLWFPHSENVSVIYQDPTNKP
Opisthocomus hoazin             TVTFQCVPQFHLHEVAFPSTLTKVLDHLEDHLKRSQYFRFLWFPHSENVSVIYQDPTNKP
Scyliorhinus torazame           NVTIQCVPAFRIHLQQFPKTLTEVLGDLDTHLKQSEYFRFFWFPHTDKVTVFYADRTNKP
                                 :* ** . * :    **.** :**..*: * ::*:****:***::: * ::* * *.*
```

```
Acipenser ruthenus              VQNTSSWFWGYAVGYHLLEFLLWVSTFLPRMVPWINQIFFSLLSSGRDERVDCSHRIFTY
Polyodon spathula               VQSTSSWFWGYAVGYHLLEFLLWVSTFLPRMVPWINQIFFSLLSSGRNERVDCSHRIFTY
Lepisosteus oculatus            AQCTSSWFWSYAVGYHLLEFVLWVSTFFPGLVCWINHLFFKLLYSGKEERIGRSDKVFTY
Amia calva                      IQSTSSWFWSYAVGYHLLQFLLWVSTFLPSLVPWLNSLFFKLLFSGREERVGRSDTIFTY
Callorhinus ursinus             PSSSATWFWDYAIGFYLLEFLLWVSSFLPGLVGWINRCFFWLLFTRKKESSNLSYKIFSY
Canis lupus familiaris          PSSSANWFWDYAIGFYLLEFLLWISSFLPGLVGWINRFFFWLLFTRKKESSDLSHKIFTY
Felis catus                     PSSSANWFWDYAIGFYLLEFLLWISSFLPGLVGWINRFFFWLLFTRKRESSNLSHEIFTY
Mus musculus                    PSSASNWFWDYAIGFYLLEFLLWTSTYLPRLVGWINRFFFWLLFNCKKESSNLSHKIFSY
Mauremys reevesii               PSSSANWFWDYAAGYYLLEFLLWISTFLPSLVCWINRFFFWLLFKGKVEHVDLSYKIFNY
Chelonoidis abingdonii          PSSSASWFWEYAVGYYLLEFLLWISTFLPGLVCWINRFFFWLLFKGKVEHVDLSYKIFNY
Alligator mississippiensis      PSSTGSWFWDYAIGYYLLEFLLWVSTFLPFLVPWINRGFFWLLFTSKGEKVDLSYKVFNY
Gallus gallus                   PSSSANWFWDYAVGYYLLEFLLWISTFVPSLVCWINRFFFWLLFSSRVENINVSYKIFNY
Meleagris gallopavo             PSSSANWFWDYAVGYYLLEFLLWISTFVPSLVRWINRFFFWLLFSSRVENINVSYKIFNY
Aquila chrysaetos               PSSSASWWWDYAVGYYLLEFLLWISTFVPSLVCWINRFFFWLLFSSRVENIAISYKIFNY
Haliaeetus albicilla            PSSSASWWWDYAVGYYLLEFLLWISTFMPSLVCWINRFFFWLLFSSRVENIAISYKIFNY
Opisthocomus hoazin             ASSSASWWWDYAVGYYLLEFLLWISTFVPGLVCWINRFFFWLLFSSRVENIAISYKIFNY
Scyliorhinus torazame           IKTTSSWFWNYAIGYYLLEFLLWISVFVPRLVPWINRLFYWLLYSAKAEQVKRSDKAFNF
                                    ..* * *. *::**:*:** * :.* :: *:*  *: ** . : .    *   *.:

Acipenser ruthenus              ECLFKQHVQDWAVPMGKTKEALLQLKAWLENNQTVVAHFPVEVRFARGDNILLSPCFQRD
Polyodon spathula               ECLFKQHVQDWAIPIGKTKEALLQLKAWLENTQAVVAHFPVEVRFARGDNILLSPCFQRD
Lepisosteus oculatus            ECLFKQHVQDWSIPMEQTKEALLQLKAWLENNPDTVAHFPVEVRFARGDNILLSPCFQRD
Amia calva                      ECLFQQHVQDWSIPIDKTKEALLQLKAWLESTPAVVAHFPVEVRFTRGDDILLSPCFQRD
Callorhinus ursinus             ECRFKQHVQDWAIPREKTKEALLELKAMLEAHPKMVAHYPVEVRFTRGDDILLSPCFQRD
Canis lupus familiaris          ECRFKQHVQDWAIPREKTKEALLELKAMLEAHPKMVAHFPVEVRFTRGDDILLSPCFQRD
Felis catus                     ECRFKQHVQDWAIPREKTKEALLELKAVLETHPKMVAHFPVEVRFTRGDDILLSPCFQRD
Mus musculus                    ECRFKQHVQDWAIPREKTKEALLELKAMLEAHPKVVAHYPVEVRFTRGDDILLSPCFQRD
Mauremys reevesii               ECRFRQHVQDWAIPIEKTKEALLELKAVLENNPKVVAHYPVEVRFARGDDILLSPCFQRD
Chelonoidis abingdonii          ECRFRQHVQDWAIPIEKTKEALLELKAVLENNPKVVAHYPVEVRFARGDDILLSPCFQRD
Alligator mississippiensis      ECRFKQHVQDWAIPIEKTKDALLELKAALESSPKVVAHYPVEVRFARADSILLSPCFQRD
Gallus gallus                   ECRFKQHVQDWAIPIEKTKEALLELKAALENNPKMVAHYPVEVRFARADEIWLSPCFQRD
Meleagris gallopavo             ECRFKQHVQDWAIPIEKTKEALLELKAALENNPKMVAHYPVEVRFARADEIWLSPCFQRD
Aquila chrysaetos               ECRFKQHVQDWAIPIEKTKEALLELKAALENNPKMVAHYPVEVRFARGDEIWLSPCFQRD
Haliaeetus albicilla            ECRFKQHVQDWAIPIEKTKEALLELKAALENNPKMVAHYPVEVRFARGDEIWLSPCFQRD
Opisthocomus hoazin             ECRFKQHVQDWAIPIEKTKEALLELKAALENNPKMVAHYPVEVRFARGDEIWLSPCFQRD
Scyliorhinus torazame           DCLFKQHVSDWALPIKQTRAALEQLKDWLDNNPNVRAHFPVEVRFVRADDILLSPCYRQD
                                :* *:***.**::*  :*: ** :**  *:      **:******.:.* * ****:::*
```

```
                                                                 HWXK Motif
Acipenser ruthenus              SCYMNIIMYRPYGKEVPRDQYWAAYEEIMQNAGGRPHWAKAHSCTRKDFQRMYPGFQKFC
Polyodon spathula               SCYMNIIMYRPYGKEVPRDQYWAAYEKIMQKVGGRPHWAKAHSCTHKNFQRMYPGFQKFC
Lepisosteus oculatus            SCYINIILYRPYGKDVPKQQYWAAYENIMKDVGGRPHWAKAHNCTRKDFEKMYPGFQKFC
Amia calva                      STYMNIIMYRPYGKDVPRQQYWAAYETIMKRVGGRPHWAKAHNCTRKDFEEMYPSFKKFC
Callorhinus ursinus             SCYMNIIMYRPYGKDVPRLDYWLTYETIMKKVGGRPHWAKAHNCTRKDFEKMYPAFSKFC
Canis lupus familiaris          SCYMNIIMYRPYGKDVPRLDYWLTYETIMKKVGGRPHWAKAHNCTRKDFEKMYPAFSKFC
Felis catus                     SCYMNIIMYRPYGKDIPRLDYWLAYETIMKKVGGRPHWAKAHNCTRKDFEKMYPAFSKFC
Mus musculus                    SCYMNIIMYRPYGKDVPRLDYWLAYETIMKKFGGRPHWAKAHNCTRKDFEKMYPAFHKFC
Mauremys reevesii               SCYMNIIMYRPYGKDVPRLDYWLAYESIMKKAGGRPHWAKAHTCTRKDFEKMYPGFRKFC
Chelonoidis abingdonii          SCYMNIIMYRPYGKDVPRLDYWLAYESIMKKAGGRPHWAKAHTCTRKDFEKMYPSFQKFC
Alligator mississippiensis      SCYMNIIMYRPYGKDVPRLDYWLAYEGIMKKFGGRPHWAKAHACTRKDFEKIYPGFPKFC
Gallus gallus                   SCYMNIIMYRPYGKNVPRLNYWLTYEGIMKKYGGRPHWAKAHSCTRKDFEKMYPAFPKFC
Meleagris gallopavo             SCYMNIIMYRPYGKNVPRLNYWLTYEGIMKKYGGRPHWAKAHSCTRKDFEKMYPAFPKFC
Aquila chrysaetos               SCYMNIIMYRPYGKNVPRLNYWLTYEGIMKKHGGRPHWAKAHSCTRKDFEKMYPAFPKFC
Haliaeetus albicilla            SCYMNIIMYRPYGKNVPRLNYWLTYEGIMKKHGGRPHWAKAHSCTRKDLEKMYPAFPKFC
Opisthocomus hoazin             SCYMNIIMYRPYGKNVPRLNYWLTYEGIMKKHGGRPHWAKAHSCTRKDFEKMYPAFPKFC
Scyliorhinus torazame           SCYINIIMYRPYGKEVPREGYWAMYEEIMKRNGGRPHWAKAHSLLRQDFEKIYPAFHKFC
                                * *:***:******:: :  **  ** **:  **********   :::::.:**.* ***
```

```
Acipenser ruthenus              SIREQLDPTGMFLNSYLEKVFF-
Polyodon spathula               SIREQLDPTAMFLNSYLEKVFF-
Lepisosteus oculatus            SIREKQDPSGVFLNTYLENIFF-
Amia calva                      SIREKLDPAGMFLNSYLEKVLIS
Callorhinus ursinus             AIREKLDPTGMFLNAYLEKVFY-
Canis lupus familiaris          AIREKLDPTGMFLNAYLEKVFY-
Felis catus                     AIREKLDPTGMFLNAYLEKVFY-
Mus musculus                    DIREKLDPTGMFLNSYLEKVFY-
Mauremys reevesii               TIREELDPTGMFLNTYLEKVFY-
Chelonoidis abingdonii          TIREELDPTGMFLNVYLEKVFY-
Alligator mississippiensis      SIREKLDPTGMFLNTYLEKVLY-
Gallus gallus                   SVRDKLDPTGMFLNTYLEKVFY-
Meleagris gallopavo             SVRDKLDPTGMFLNTYLEKVFY-
Aquila chrysaetos               SVREKLDPTGMFLNTYLEKVFY-
Haliaeetus albicilla            SVREKLDPTGMFLNTYLEKVF--
Opisthocomus hoazin             AVREKLDPTGMFLNAYLEKVFY-
Scyliorhinus torazame           SIREELDPSGMFLNNYLEKTFF-
                                 :*:  **:.:*** ***: :
```

Figure S6. Multiple alignments of the GULO amino acid sequence with known eukaryotic GULO protein homologs. The GenBank accession numbers of the GULO protein homologs are as follows: *Acipenser*

ruthenus, RXM28160.1; *Polyodon spathula*, XP_041109140.1 ; *Lepisosteus oculatus*, XP_015207781.2 ; *Amia calva*, XP_066566221.1 ; *Callorhinus ursinus*, XP_025729573.1; *Canis lupus familiaris*, XP_005635730.2; Felis catus, XP_019684152.1 ; *Mus musculus*, NP_848862.1 ; *Mauremys reevesii*, AET14635.1 ; *Chelonoidis abingdonii*, XP_032657598.1 ; *Alligator mississippiensis*, XP_014453746.3; *Gallus gallus*, XP_015140704.1 ;*Meleagris gallopavo*, XP_003204615.2 ; Aquila chrysaetos, XP_040984784.1 ; *Haliaeetus albicilla*, NWZ47405.1; *Opisthocomus hoazin*, XP_009936988.1 ; *Scyliorhinus torazame*, Q90YK3.1.
